# Late-onset jaw and teeth pain mimicking trigeminal neuralgia associated with chronic vagal nerve stimulation: case series and review of the literature

**DOI:** 10.1186/s12883-017-0892-4

**Published:** 2017-06-15

**Authors:** Gabriela Timarova, Andrej Šteňo

**Affiliations:** 1 2nd Department of Neurology, Faculty of Medicine, Comenius University, Dérer’s University Hospital, Limbova str.5, 83305 Bratislava, Slovak Republic; 2 Department of Neurosurgery, Faculty of Medicine, Comenius University, Dérer’s University Hospital, Bratislava, Slovak Republic

**Keywords:** VNS, Epilepsy, Trigeminal pain, Side effects, Pain, Case report

## Abstract

**Background:**

Vagal nerve stimulation (VNS) for refractory epilepsy is well established. Trigeminal neuralgia itself is a common disease in adults, and thus, late-onset pain in the trigeminal region under VNS, which is extremely rare, may not be recognized as caused by VNS.

**Case presentation:**

Two patients with drug-resistant symptomatic epilepsy treated with chronic VNS experienced stimulation-related pain in the lower and upper jaw and teeth on the side of stimulation. No evidence of local spread of the stimulation current was present. The pain started with a delay of years after device implantation and weeks after the last increase in the pacing parameters. At the time of onset, the pain was not recognized as VNS-related, leading to extensive examinations. The trigeminal neuralgia-like pain resolved after adjustment of the stimulation current intensity. In one of the patients, the pain disappeared within one to two days following every epileptic seizure. To our knowledge, this is the first case report of late-onset trigeminal pain under VNS revealing a direct link between epileptogenic and pain processes.

**Conclusion:**

A painless interval between the last change of the pacing parameters and trigeminal pain can lead to the erroneous interpretation that this is a typical trigeminal neuralgia. The lack of its recognition as a side effect of VNS can lead to unnecessary examinations and delayed adjustment of stimulation parameters. In patients with signs of late-onset trigeminal pain under VNS with normal electrode impedance and no evidence of local current spread, the replacement of the VNS lead does not seem to be beneficial. A review of the literature on VNS side effects including pain and device malfunctions was undertaken.

## Background

Vagal nerve stimulation (VNS), delivered by the NCP System (Cyberonics, Houston, TX, USA) for treatment of drug-resistant epilepsy is approved as an add-on therapy in adults and children for partial and generalized epileptic seizures. New, noninvasive stimulation devices are under development [1, 2]. The VNS efficacy has been established, showing a 50% reduction in epileptic seizure rate in approximately 30% of patients after one year with an increase to approximately 50-70% after three years, with relatively few patients (less than 10%) becoming seizure free [3–5]. Despite more than 20 years of VNS accessibility, the discussion of its safety and efficacy is ongoing. The evidence-based guidelines from the American Academy of Neurology in 2013 [6] emphasized the need for further safety information.

The adverse events (AE) of VNS are of two types: implantation procedure-related and stimulation-related. Surgery-related AE have been reported in 3-22% of VNS implantations. The most often reported surgery-related AE are hardware failure in 3.7-16.8%, lead fracture or disconnection in 3.7-13.7%, wound infections in 1.7-7.1%, wound hematoma in 0.7-1.9%, transient asystole/bradycardia up to 1%, left vocal cord palsy, mostly transient, in 1.4-5.1%, and lower facial weakness in 0.2-1.2% [7–14]. Stimulation-related AE in different studies have been reported to occur in up to 68% of patients, with 97.8% of the AE reported as mild to moderate. The AE usually appeared immediately after VNS adjustments and disappeared spontaneously over some time or after the adjustment of the stimulation current to the previous level of stimulation [7, 15–17]. Most often reported stimulation-related AE were voice alterations (6-66%), hoarseness (1.4-64%), cough (7-45%), dyspnea (2-25%), throat pain (4.7-22%), neck pain and/or tingling and twitching in the neck muscles (0.5–22%), dysphagia (13-17.9%), headache (7-30%) and chest pain (up to 13%). Cases with some pain were reported in 6-30% of implantations [7, 18–23]. In addition to the VNS side effects reported in population studies, there are rare cases or case series reports of unusual or late-onset stimulation-related AE such as parkinsonism [24], late-onset bradyarrhythmia/asystole [25–28], sleep apnea [29, 30], psychosis or mania [31], glossopharyngeal tonsillar pain [32] and pharyngeal dysesthesia [33]. Cases of late-onset trigeminal pain associated with VNS, considering the large number of VNS implantations performed worldwide, are an extremely rare and unexpected event [34, 35] (Table 1).

**Table 1:** Characteristics of late-onset trigeminal pain under VNS in reported patients

Author	Type of disease	Time to pain onset from implantation	Time to pain onset from last augmentation	SC	Pain localisation
				(mA)	
Shih [35]	Epilepsy-Tuberous sclerosis	9 months	2 months	1.25^a^	Left cheek, mentally retarded child with unprecise description of the pain
Carius and Schulze-Bonhage [34]	Cryptogenic epilepsy, focal seizures	5 months	few days	1.5^a^	The lower jaw, left
	Epilepsy-right frontotemporal	2 months	1 month	0.5^a^	The lower jaw and occipital headache, left
	Epilepsy- bitemporal	11 months	2 weeks	1.75^a^	The lower jaw and throat, left

*SC* stimulation current intensity at the time of pain onset

^a^ In all patients was stimulation frequency 30 Hz, pulse width 500usec, duty cycle 30s on and 5 min off

## Case presentation

At the University Hospital Bratislava, Slovakia, we implanted VNS systems in 54 patients with drug-resistant epilepsy not amenable to resection epilepsy surgery between April 2009 and December 2016. Forty-seven of the patients were followed for a long time (one to eight years). The VNS systems were implanted on the left side, and patients had regular follow-up visits with a stepwise increase in stimulation current by 0.25 mA in 4-8 weeks. The target range of the stimulation current intensity, if tolerated, was between 1.25 and 2.00 mA with a stimulation frequency 25-30 Hz, pulse width of 250-500 μsec and a duty cycle of 30-21 s on and 5 to 1.1 min off, as recommended by the manufacturer.

Two of our patients perceived stimulation-related pain in the upper and lower jaw and teeth ipsilateral to the side of stimulation with a delay of years after device implantation and weeks after last augmentation of the stimulation current intensity, thus mimicking coincidental trigeminal neuralgia to the VNS (Table 2). Both were treated with antiepileptic drugs (AED), which are usually effective in pain treatment (see below), but the AED were not effective in the prevention and control of the pain associated with VNS stimulation.

**Table 2:** Characteristics of late-onset trigeminal pain under VNS in the current case series patients

Sex (age, years)	Type of epilepsy	Time to pain onset from implantation	Time to pain onset from last augmentation	SC	Pain localisation
				(mA)	
Man (46)	Bitemporal-periventricular heterotopy	18 months	2 months	2,0^a^	The upper and lower jaw and teeth, left
Woman (50)	Bitemporal- bilateral cystic hippocampal malformations	4,5 years	2 weeks	1,25^b^	The upper and lower jaw and teeth, left

*SC* stimulation current intensity at the time of pain onset

^a^ Stimulation frequency 30 Hz, pulse width 500 μsec, duty cycle 30s on/ 1,8 min off

^b^ Stimulation frequency 30 Hz, pulse width 250 μsec, duty cycle 30s on/ 1,8 min off

## Case 1

A 46-year-old man with intractable symptomatic bitemporal epilepsy lasting 33 years, with MRI-verified right temporooccipital periventricular heterotopy, was implanted with VNS in November 2012. His seizure frequency before implantation was up to 10 motor seizures with impaired awareness per month and sporadic bilateral tonic-clonic seizures. After implantation, the stimulation current was increased stepwise in 4-8 weeks. He achieved a 50% reduction in seizures with 2.0-mA stimulation current, 30-Hz frequency, 500-μsec pulse width, and 30-s on-time and 1.8-min off-time. Eighteen months after initiation of VNS stimulation, two months since the last increase in the stimulation current, the patient began to complain of sharp, shooting pain in the upper and lower jaw and teeth on the left side, without trigger points or a sensory deficit. The patient underwent CT scans and detailed dental examination, but no pathological processes were discovered. It was the patient who first noticed that the painful shootings were regular, lasting for tens of seconds with the stimulation on, and that the painless intervals lasted minutes with the stimulation off. The consequent analysis, when we checked the painful and pain-free intervals, ascertained that the pain was associated with the stimulation period of VNS. When the device was off, he was pain-free. The impedance of the electrode was normal. The intensity of the pain was evaluated as 10 on a visual analogous 10-point scale (VAS). The patient had no signs of local current spread to the surrounding tissues (breathing and voice problems, muscle tingling or twitching, no pain in the head, chest or neck). The pain mimicked trigeminal neuralgia type 1 according to Burchiel’s classification [36]. According to the seizure diary, where the patient recorded all seizures and painful days, there was a discontinuous course of pain attacks. He was pain-free for 1-2 days following every epileptic seizure, with reoccurrence of the pain in the following days. His antiepileptic treatment that time was a combination of lacosamide at 400 mg/day, lamotrigine at 200 mg/day and pregabaline at 300 mg/day. He was treated for concomitant hypertension with perindopril at 4 mg/day, moxonidine at 0.2 mg/day and rilmenidine at 1 mg/day and for anxiety with alprazolam at 1 mg/day. We started adjustments of the stimulation parameters of VNS with a three-month delay due to diagnostic work-up. The patient reported pain relief at a stimulation current of 1,5 mA and complete resolution at a stimulation current of 0.5 mA. At that time, the frequency of the seizures increased to the original level with a loss of responsivity. At that time, the patient preferred to switch off the device. Six months later, the system was retested. He tolerated a stimulation current up to 1.5 mA without painful sensations.

## Case 2

A 50-year-old woman with intractable symptomatic bitemporal epilepsy lasting 37 years, with MRI-verified bilateral cystic malformations of mesial temporal lobe structures, was implanted with VNS in April 2011. Her seizure frequency was up to 6 motor seizures with impaired awareness per month and sporadic bilateral tonic-clonic seizures. After VNS implantation, the stimulation current was increased stepwise in 4-8 week intervals up to a 1.0-mA stimulation current intensity, 30-Hz frequency, 250-μsec pulse width, 30-s on-time and 3-min off-time. At these stimulation parameters, she was a 50% responder. Four years later, she overcame status epilepticus. The stimulation current was increased to 1.25 mA and the duty cycle to 30 s on and 1.8 min off. Two weeks later, she began to complain of shooting, sharp pain in the upper and lower jaw and teeth on the left side, without a trigger point or sensory deficit. The intensity of the pain was evaluated as an 8 on the VAS. The pain mimicked trigeminal neuralgia type 1 according to Burchiel’s classification [36]. The patient had no signs of local current spread to surrounding tissues. At that time, she was treated with anti-epileptic drugs levetiracetam at 2000 mg/day, lamotrigine at 300 mg/day and carbamazepine at 1200 mg/day. She did not use any other drugs or treatments. The impedance of the electrode was normal. Because of the experience with patient 1, we immediately checked the relation of the shooting pain to the stimulation period of the VNS and the relation of the pain-free intervals to the off period of the VNS. The pain was recognized as stimulation-related, and we immediately began the adjustments. The patient reported complete pain resolution at a 1.0-mA stimulation current and continued the VNS.

## Discussion and conclusions

Carius and Schulze-Bonhage [34] reported late-onset trigeminal pain in 3 out of 27 implanted patients (11.1%), whereas we found it in 2 out of 47 implanted patients longer than one year after implantation (4.3%). Carius and Schulze-Bonhage proposed mechanisms of central sensitization as the probable cause of the reported pain [34]. Later, Spitz et al. [37] reported a case with a small discontinuity in the lead silicone insulation that led to vocal cord paralysis, impaired breathing and cervical, mandibular, pharyngeal and dental pain. The electrode impedance was normal. The problems started in the early titration period, and the maximum tolerated stimulation current intensity was low (0.5 mA maximum). Spitz et al. [37] postulated that the aberrant spread of current through the disrupted insulation likely accounted for other reports of stimulation-related pain (referred trigeminal pain, tonsillar pain, sometimes delayed onset). In other cases, where device malfunction was confirmed, clinical signs of the spread of the stimulation current to surrounding tissues were described (hoarseness, vocal cord palsy, distorted breathing, pharyngeal and neck pain, tingling or twitching in the neck muscles and diaphragm dysfunction). The problems typically started after an accident (trauma, puncture, traction, excessive manipulations, tight electrode). In most cases of device malfunctions, the lead impedance is too high or too low [38–41].

In our patients with late-onset jaw and dental pain, the gradual increase in the stimulation current intensity up to 1 mA and more was uncomplicated. The pain appeared after a pain-free interval from the last adjustment without any accident or trauma and no signs of local current spread. After a 6-month stimulation-free period, patient 1 regained the tolerance for stimulating current up to 1.5 mA, thus pointing to possible desensitization. A unique, intermittent course of stimulation related pain was documented in him with pain relief for 24-48 h following every epileptic seizure. Mechanisms of activity-dependent central sensitization are likely explanations [42]. The nucleus of the solitary tract is the recipient of most afferent sensory fibers of the vagal nerve, but the vagal nerve also sends ipsilateral projections to the spinal trigeminal nucleus (STN). Animal studies have revealed an interesting pattern of trigeminal nociceptive neuronal activation and somatic-visceral trigemino- vagal integration that is mediated by vagal afferents to STN. Central sensitization has been described in the dorsal horn of the spinal cord, as well as in the STN pars caudalis (Vc) and the transition zone (Vi/Vc) [43]. In an animal experiment, activation of vagal C-fibers was confirmed to not be required to obtain VNS-induced seizure suppression; activation of A- and/or B-fibers seems to be sufficient. These data are clinically important since A- and B-fibers have a much lower activation threshold than C-fibers, thus reducing the amount of current necessary to produce the antiepileptic effects of VNS. Lack of C-fiber recruitment is also important since activation of these fibers would produce central sensitization and undesirable side effects that are not seen in most patients and may have rendered the therapy intolerable in some [44]. The modulatory effects of vagal nerve stimulation on nociception have been studied in animal studies, including the effects in the STN. Both facilitatory and inhibitory effects on neuronal responses to noxious stimuli were observed [45, 46]. The stimulation parameters favoring pro- or antinociceptive effects of VNS in man are not known [42]. Postictal pain relief was observed in one of our cases. To the best of our knowledge, this is the first case report of late-onset trigeminal pain under VNS revealing a direct link between epileptogenic and pain processing. The postictal state is generally followed by antinociception. Intrinsic neural circuits between dorsal midbrain neurons control seizure activity and the nuclei of the pain inhibitory system elaborating postictal antinociceptive processes. Endogenous opioid-, acetylcholine-, serotonin-, and norepinephrine-mediated systems have been implicated in the organization of tonic-clonic seizure-induced anti-nociception [47]. The locus coeruleus represents a key structure in the organization of epilepsy-induced norepinephrine-mediated hypoalgesia, and its lesions suppress the seizure-attenuating effects of VNS [48–50].

With reference to the data accumulated in previous years, late-onset trigeminal pain under VNS stimulation in our patients and patients reported previously can be explained by mechanisms of activity-dependent central sensitization, lead revisions in cases with normal electrode impedance and no signs of local spread of the current seem not to be beneficial. It has to be recognized by physicians, so immediately began to reduce the stimulation current intensity.

## Abbreviations

AE: Adverse events
AED: Antiepileptic drug/drugs
CT: Computed tomography
MRI: Magnetic resonance imaging
STN Vc: Spinal trigeminal nucleus pars caudalis
STN Vi/Vc: Spinal trigeminal nucleus transition zone
STN: Spinal trigeminal nucleus
VAS: Visual analogous scale
VNS: Vagal nerve stimulation
